# Improvement of Photosynthesis by Biochar and Vermicompost to Enhance Tomato (*Solanum lycopersicum* L.) Yield under Greenhouse Conditions

**DOI:** 10.3390/plants11233214

**Published:** 2022-11-24

**Authors:** Xinna Liu, Jie Zhang, Qian Wang, Tingting Chang, Hiba Shaghaleh, Yousef Alhaj Hamoud

**Affiliations:** 1 College of Agricultural Science and Engineering, Hohai University, Nanjing 210098, China; 2 College of Environment, Hohai University, Nanjing 210098, China

**Keywords:** biochar, vermicompost, net photosynthetic rate, intercellular CO_2_ concentration, photosystem II, maximum photochemical efficiency, active reaction center density

## Abstract

Chlorophyll fluorescence is an important tool in the study of photosynthesis and its effect on the physiological indicators of crop growth is worth exploring. The trial was conducted to investigate the effect of biochar (CK, 0%; BA_3_, 3%; BA_5_, 5%; by mass of soil) and vermicompost (VA_3_, 3%; VA_5_, 5%) on photosynthesis, chlorophyll fluorescence, and tomato yield under greenhouse condition. Results revealed that photosynthetic parameters and chlorophyll fluorescence traits of BA_3_, VA_3_, BA_5_, and VA_5_ were significantly higher than those of CK, and the improvement of vermicompost was more effective than biochar at the same application rate. VA_3_ treatment had the highest net photosynthetic rate (*Pn*), intercellular CO_2_ concentration (*Ci*), variable fluorescence (*Fv*), maximum fluorescence (*Fm*), *PSII* maximum photochemical efficiency (*Fv/Fm*), *PSII* potential photochemical activity (*Fv/Fo*), absorption flux per cross section (CS; *ABC/CSm*), trapped energy flux per CS (*TRo/CSm*), and electron transport flux per CS (*ETo/CSm*), which increased by 49%, 65%, 17%, 12%, 4%, 25%, 10%, 15%, and 30%, respectively, compared with CK. The study also found that BA and VA rates could effectively improve tomato yield and water use efficiency (*WUE*). The yield under BA_3_, VA_3_, BA_5_, and VA_5_ treatments was 21%, 33%, 23%, and 25% higher than that under CK, and the *WUE* increased from 31.2 kg·m^−3^ under CK to 41.4 kg·m^−3^ under VA_3_. Pearson correlation analysis indicated that the increment of photosynthesis showed a highly significant correlation with *Fv/Fo*, *ABC/CSm*, *TRo/CSm*, and *ETo/CSm* and enhanced the light energy absorbed, trapped, and transported per CS of plant leaves, thereby contributing to the increase in tomato yield. Therefore, for one-season tomato production, the application of 3% vermicompost was considered economical with regard to improving photosynthesis, enhancing *WUE*, and increasing tomato yield.

## 1. Introduction

As an effective way to improve vegetable production, facility cultivation plays an important role in anti-seasonal and inter-regional vegetable cultivation in China [1]. Tomato is a temperature-loving, light-loving, and semi-drought-tolerant facility cultivation crop with high promotion potential and economic value [2]. However, unreasonable fertilizer application and cultivation management have resulted in facility soils showing a susceptibility to pests and diseases, which seriously affected the growth and development of tomatoes and reduced tomato yield [3,4]. Therefore, optimizing the fertilizer application pattern and improving the photosynthesis and chlorophyll fluorescence characteristics of plants are important for the high yield and quality of tomatoes.

Photosynthesis is the basic physiological activity for crop yield formation, and the strength of photosynthesis is closely related to the level of yield [5,6,7]. Photosynthesis is influenced by various environmental factors, including water, nutrients, light, and CO_2_ concentration [8,9,10]. Biochar is beneficial to plant growth and physiological characteristic indicators, and it can improve the growth performance and yield of different crops [11]. Appropriate application of biochar can improve the apparent quantum efficiency, *Pn*, photosynthetic capacity, and stomatal conductance (*Gs*) of plants [12], which can play a role in improving crop quality and yield [13]. Zhu [14] showed that biochar increased *Pn* and seedling emergence and promoted plant height and dry matter mass of tomatoes. Cao et al. [15] found that biochar improved the nutritional quality of cherry tomato fruits and increased the yield. Lu et al. [16] concluded that biochar could increase the chlorophyll content of plant leaves and positively affect crop yield. The special physical and chemical properties and biological structure of vermicompost improve the transpiration rate (*Tr*), *Gs*, *Pn*, and intercellular CO_2_ concentration (*Ci*) of tomato leaves, thereby promoting photosynthesis in plants [17]. Hosseinzadeh et al. [18] showed that the application of vermicompost could improve the photosynthesis of the crop primarily because it increased the CO_2_ content of the crop roots and improved the soil water-holding capacity. In addition, numerous studies have demonstrated that the application of vermicompost in soil had a positive effect on crop growth and yield [19]. Zhou et al. [20] showed that the application of 80% vermicompost in soil significantly increased the growth of height and stem diameter of tomato plants. Joshi et al. [21] revealed that the application of 45% vermicompost also promoted the growth and development of tomato plants.

Geng et al. [22] concluded that chlorophyll fluorescence parameters could reflect the absorption and conversion of light energy, energy transfer, distribution, and photosynthesis in plants, which were important indicators used to study plant stress resistance physiology and increase crop yield. Chlorophyll fluorescence can be used to study the effect of environmental changes on the photosynthetic structure of plant photosystem II (*PSII*) and yield responses to the efficiency of light energy conversion in plants [23,24]. Li et al. [25] revealed that biochar treatment could significantly affect the chlorophyll fluorescence parameters of cucumber, but the application of 0.5–2.0% biochar had no significant effect on the maximum photochemical efficiency (*Fv/Fm*). Meanwhile, Cheng et al. [26] also revealed that *Fv/Fm* and the actual quantum yield of *PSII* (*Y (II)*) of plant leaves significantly increased when the vermicompost content was higher than 50%. Gong et al. [27] found that *Fv/Fm* of *PSII* and the actual photochemical efficiency (*ΦPSII*) of plants were elevated by water–nitrogen content, and a moderate increase in nitrogen fertilization could improve *Fv/Fm* and *ΦPSII* of crop leaves, enhance crop growth traits, and increase yield.

Numerous studies have shown that the application of biochar and vermicompost changed the physicochemical properties of soil and microbial communities. It also affected the physiological and biochemical properties of plants. Furthermore, photosynthesis has become an important indicator of tomato production, which is essential for promoting plant growth and development and improving yield. Therefore, the tomato water use efficiency (*WUE*) together with plant physiology must be improved through the use of accurate soil management methods, with emphasis on the methods that improve soil quality by the application of biochar and vermicompost with a considerable enhancement in plant physiological responses and yield. To date, there has been little knowledge on the interactive effects of biochar and vermicompost application on photosynthesis rate, especially using the chlorophyll fluorescence of tomato as a probe. Additionally, the effects of biochar and vermicompost on synergistic response of plant growth are not well understood. Moreover, data on the synergistic response of tomato yield with *WUE* are largely scarce [28]. Thus, this study hypothesized that increasing biochar and vermicompost addition could improve the soil properties; regulating the plant photosynthesis by improving chlorophyll fluorescence parameters would, thus, improve the tomatoes’ productivity. The study also assumed that the biochar and vermicompost application to the plant can increase plant growth, contributing to an increasing yield of tomato. To test the hypothesis mentioned above, the study investigated the effects of increasing biochar and vermicompost amendment application rates under greenhouse conditions on soil properties related to plant growth. The study also measured the photosynthesis and chlorophyll fluorescence of tomato by measuring the *Pn*, *Tr*, *Gs*, *Ci*, including *Fo*, *Fv*, *Fm*, *Fv/Fm*, *Fv/Fo*, *ABC/CSm*, *RC/CSm*, *ETo/CSm*, *DIo/CSm*, and *TRo/CSm* and, thus, regulated tomato yield in a greenhouse experiment to provide proper regulation for the high quality and yield of tomatoes under greenhouse conditions.

## 2. Materials and Methods

### 2.1. Experimental Site

Greenhouse experiments were carried out from 25 July 2020 to 11 January 2021 in a non-temperature-controlled greenhouse under natural light conditions, at the water-saving Park of Hohai University located at latitude 31°57′ N and longitude 118°50′ E, at 144 m above sea level in Jiangning District, Nanjing City, Jiangsu Province, China. The climate of the region is humid subtropical, and it is influenced by the East Asian monsoon. The average annual temperature in the region was 15.7 °C; the absolute maximum temperature reached 40.4 °C in August 2020, and the absolute minimum temperature dropped to −13.3 °C in January 2021. The rainy season spanned from July to September, and the average annual rainfall in the area was nearly 1025.12 mm, which was concentrated in the rainy–summer season. The annual sunshine time was 2200 h, and the annual average evaporation was approximately 900 mm. The average monthly rainfall and temperature in the greenhouse during the years of the experiment (2020–2021) are shown in Table 1.

### 2.2. Soil, Biochar, and Vermicompost Preparation

The experimental soil was collected from the top 10–20 cm of the farmland soil of the water-saving Park of Hohai University (31°57′ N, 118°50′ E) and classified as a typical yellow–brown loam based on the Chinese classification [29]. The tested biochar was classified as maize straw biochar (purchased from Henan Lize Environmental Protection Technology Co., Ltd., Zhengzhou, China), and the experimental vermicompost was obtained by fermenting pure cow dung through the digestive system of earthworms. The physicochemical properties of the soil before the experiment, biochar, and vermicompost are shown in Table 2.

The physicochemical properties of the abovementioned soil samples, biochar, and vermicompost were measured by the following methods: Available potassium was determined using flame photometry [30]. Available nitrogen was determined by using a UV–Vis spectrophotometer (L007, 7522112059A; Essence Technology Instruments, Shanghai, China) [31,32]. Available phosphorus was measured using UV–Vis spectrophotometry [33]. Organic matter content was determined using high-temperature oxidation [34]. pH value was determined by using the Remag pH meter in 1:5 samples and water extracts [35].

### 2.3. Greenhouse Experimental Setup

**Figure 1:**
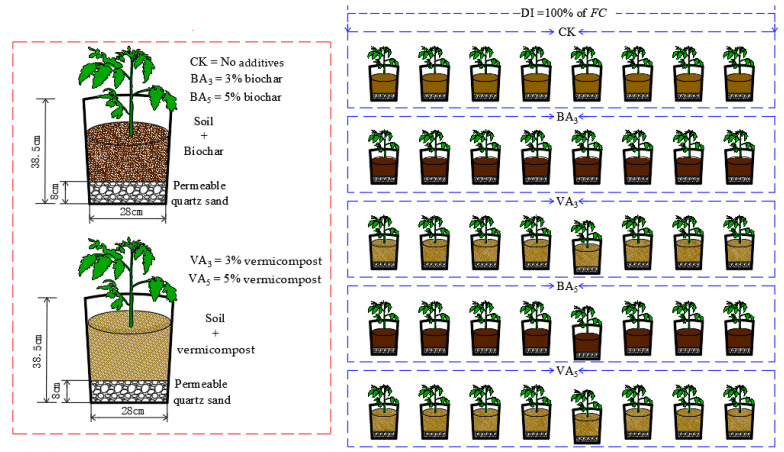
Layout of tomato pots in the greenhouse.

The main treatments used biochar and vermicompost. This experiment included the following five treatments: CK (0% rate, 12 kg soil + no addition), BA_3_ (3% rate, 12 kg soil + 360 g biochar), VA_3_ (3% rate, 12 kg soil + 360 g vermicompost), BA_5_ (5% rate, 12 kg soil + 600 g biochar), and VA_5_ (5% rate, 12 kg soil + 600 g vermicompost) on a mass basis. Each pot (cylindrical, top diameter: 32.5 cm, bottom diameter: 28 cm, height: 38.5 cm) was filled with quartz sand to a height of 8 cm, considering the water permeability and air permeability of the roots. The soil was air-dried and passed through a 6.3 mm sieve, and then biochar and vermicompost were weighed and mixed with soil thoroughly in proportion, respectively, and added to the pot with a natural bulk density based on each treatment. The pots were placed in a non-temperature-controlled greenhouse under natural light conditions and arranged in a completely randomized block design. Each treatment was replicated eight times (Figure 1).

The experimental tomato variety was “Cooperative 903,” which is a widely cultivated vegetable planted in Jiangsu Province, China. Tomato seeds were sown at a density of 2–3 seeds per hole in a 72-hole plate of cultivation seedlings and diluted to one plant per well after 2 weeks of seed germination. When seedlings developed five leaves and a heart, seedlings of similar growth were selected and transplanted into pots on 25 August 2020. The tomatoes were managed uniformly based on the experience of local agronomic practices. That is, each pot was applied with 20 g of compound fertilizer (N:P:K = 15:15:15) as a base fertilizer. Each pot was irrigated with tap water to maintain the soil water content at *FC*. The irrigation of each pot was carried out in accordance with the difference in daily weight to compensate for the water loss caused by evaporation [36], and the soil moisture content of all pots was maintained at *FC* throughout the experimental period. Each pot was frequently weeded by hand, and four fruits were left on each inflorescence in each treatment, leaving three leaves pinched at the top after the second inflorescence had set fruit. In addition, field management was carried out in the greenhouse to control pests and diseases and avoid yield losses. The final harvest was completed in January 2021.

### 2.4. Measurement Items and Methods

#### 2.4.1. Determination of Photosynthetic Parameters

During flowering and fruit setting of the experiment, four plants were randomly selected for each treatment. Healthy fully expanded leaves with sufficient light exposure and consistent leaf position and without visible symptoms of damage at the first inflorescence were selected from each plant. The net photosynthetic rate (*Pn*), stomatal conductance (*Gs*), transpiration rate (*Tr*), and intercellular CO_2_ concentration (*Ci*) were determined using a portable photosynthesis system (Li-6800, LI-COR, Lincoln, NE, USA) under an artificial light source with a radiation flux density of 1000 µmol·m^−2^·s^−1^ from 9:00 a.m. to 11:00 a.m. on a sunny day. The limitation of stomatal conductance (*Ls*) was calculated using the following equation [37]:

(1) $$Ls=1-\frac{Ci}{Ca}$$

where *Ls* is the limitation of stomatal conductance; *Ci* is the intercellular CO_2_ concentration; *Ca* is the ambient CO_2_ concentration.

#### 2.4.2. Determination of Chlorophyll Fluorescence Traits

Chlorophyll fluorescence parameters of plant leaves were measured using a portable chlorophyll fluorometer (Pocket PEA, Hansatech, King’s Lynn, UK), and the measurement time and plant site were the same as the photosynthetic parameters. After the leaves were subjected to dark-adapted treatment for 20 min, the rapid chlorophyll fluorescence induction kinetic curve (O-J-I-P curve) was measured using Pocket PEA, which was induced by 5000 µmol·m^−2^·s^−1^ of pulsed light, and the fluorescence signal was recorded from 10 μs to 2 s. The initial rate of recording was 105 datapoints per second, and the initial fluorescence (*Fo*), maximum fluorescence (*Fm*), and fluorescence intensity at 2 ms of the O-J-I-P curve (*F_J_*) were obtained. Fluorescence parameters were calculated as follows [38,39]: variable fluorescence (*Fv* = (*Fm* − *Fo*)/*Fm*), *PSII* maximum photochemical efficiency (*Fv/Fm*), and *PSII* potential photochemical activity (*Fv/Fo*). In addition, the tomato leaf energy partitioning ratio and *PSII* reaction center activity parameters were measured as follows: absorption flux per cross section (*ABC/CSm* ≈ *Fm*), trapped energy flux per CS (*TRo/CSm* = (1 − *Fo*/*Fm*)·(*ABC/CSm*)), electron transport flux per CS (*ETo/CSm* = (1 − *Fo*/*Fm*)·(1−(*F_J_* − *Fo*)/(*Fm* − *Fo*)·(*ABC/CSm*)), non-photochemical quenching per CS (*DIo/CSm* = (*ABC/CSm*) − (*TRo/CSm*)), and the number of active reaction centers per CS (*RC/CSm*) [40].

#### 2.4.3. Determination of Tomato Yield and WUE

At the mature stage of tomato, the yield of four plants selected for each treatment was measured. Fruits of two inflorescences were collected sequentially on the basis of their ripeness and weighed on an electronic scale with an accuracy of 0.01 g. Then, the total fresh weight of fruits of these two inflorescences was calculated as the total yield of each plant. The yield was converted on the basis of planting density (45,000 plants·hm^−2^). The WUE was determined using the following equation [41]:

(2) $$WUE=\frac{Y}{TWU}$$

where WUE is the water use efficiency, kg·m^−3^; Y is the tomato yield, kg·hm^−2^; and TWU is the total water use, m^3^·hm^−2^.

### 2.5. Data Processing and Analysis

Experimentally measured data were recorded and analyzed by Excel 2010 and one-way analysis of variance (ANOVA) and plotted by GraphPad Prism 8.0. ANOVA was performed using SPSS 26.0, where Duncan’s multiple range test was used to compare data means at the 0.05 level of significance, and statistical significance was considered when *p* ≤ 0.05. Pearson correlation analysis was also conducted to obtain the degree of relationship among photosynthetic parameters, chlorophyll fluorescence traits, and tomato yield.

## 3. Results and Analysis

### 3.1. Net Photosynthetic Rate and Photosynthetic Parameters

**Figure 2:**
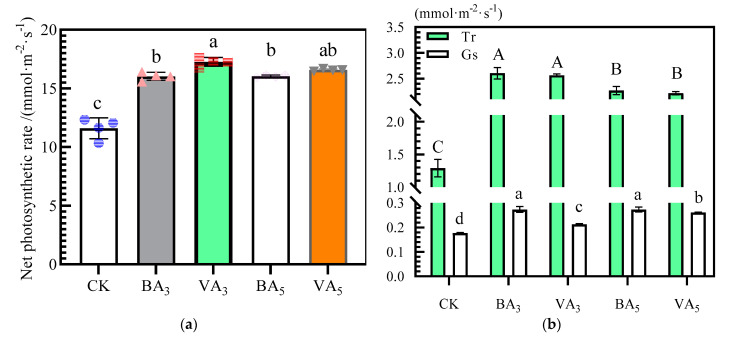

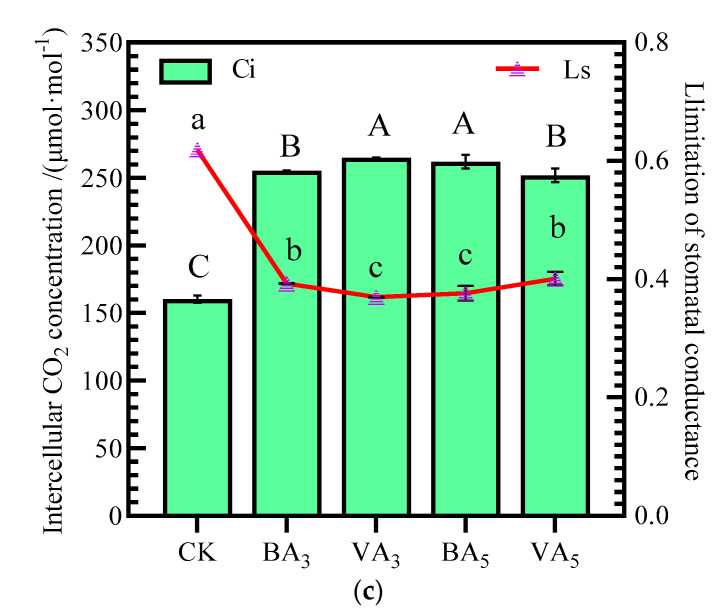
Effects of BA and VA rates on photosynthetic parameters of tomato leaves: (**a**) Effects of BA and VA rates on the *Pn.* (**b**) Effects of BA and VA rates on the *Tr* and *Gs*. (**c**) Effects of BA and VA rates on the *Ci* and *Ls.* Note: BA represents biochar application and VA vermicompost application. The meanings of the circles, triangles, and rectangles on the bars in (**a**) are indicated as duplicate data points for the different treatments. Means of *Pn*, *Gs*, and *Ls* are significantly different between BA and VA rates (*p* ≤ 0.05) when followed by different lowercase letters. Means of *Tr* and *Ci* are significantly different between BA and VA rates (*p* ≤ 0.05) when followed by different uppercase letters.Meanwhile, the increase in *Pn* in BA_3_, VA_3_, BA_5_, and VA_5_ was accompanied by a decrease in *Ls* and an increase in *Ci*, *Tr*, and *Gs*. As shown in (**b**), the highest and lowest *T_r_* rates were observed for BA_3_ and CK treatments, respectively, and BA_3_ and VA_3_ showed no statistically significant difference. *G_s_* under BA_3_ treatment was the highest, whereas that under CK treatment was the lowest. For *C_i_* (**c**), the highest *C_i_* was observed for treatments under VA_3_, followed by plants under BA_5_, BA_3_, and VA_5_, whereas the lowest values were observed under CK. *L_s_* under BA_3_, VA_3_, BA_5_, and VA_5_ treatments decreased by 37%, 40%, 39%, and 35%, respectively, compared with that under CK treatment.

The measured plant photosynthetic parameters during flowering and fruit setting of the experiment were significantly (*p* < 0.05) affected by biochar and vermicompost application (Figure 2). *Pn* increased significantly with the increase in BA and VA rates (Figure 2a), in which VA_3_ treatment had the highest *Pn*, with an increase of 49% compared with CK. In addition, VA_3_ was significantly different from BA_3_ and BA_5_ (*p* < 0.05), and no significant difference was observed between BA_3_ and BA_5_ treatments.

### 3.2. Chlorophyll Fluorescence Traits

The important chlorophyll fluorescence parameters of *Fo*, *Fv*, *Fm*, *Fv/Fm*, and *Fv/Fo* for different biochar and vermicompost addition rates during flowering and fruit setting are shown in Table 3. *Fv*, *Fm*, *Fv/Fm*, and *Fv/Fo* under BA_3_, VA_3_, BA_5_, and VA_5_ treatments, respectively, were significantly increased compared with those under CK treatment (*p* < 0.05). The highest *Fv*, *Fm*, *Fv/Fm*, and *Fv/Fo* were observed after VA_3_ treatment, which increased by 17%, 12%, 4%, and 25%, respectively, compared with CK, where *Fv* and *Fv/Fo* of VA_3_ were significantly higher than other treatments. On the contrary, BA_3_ and BA_5_ showed no statistically significant difference.

**Figure 3:**
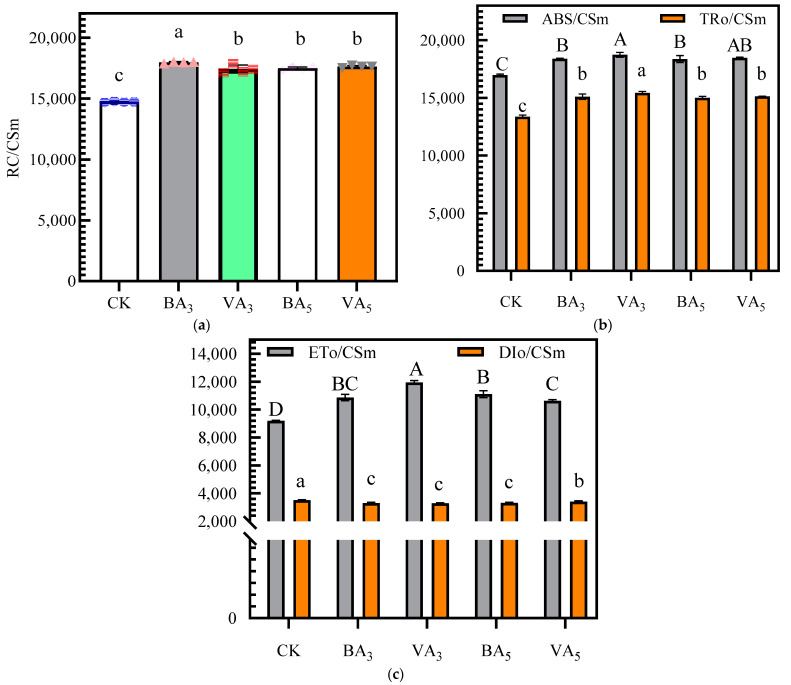
Effect of BA and VA rates on light energy absorption, capture, and transfer: (**a**) Effect of BA and VA rates on the *RC/CSm.* (**b**) Effect of BA and VA rates on the *ABC/CSm* and *TRo/CSm*. (**c**) Effect of BA and VA rates on the *ETo/CSm* and *DIo/CSm*. Note: BA represents biochar application and VA vermicompost application. Data are presented as mean ± standard error (n = 4). The meanings of the circles, triangles, and rectangles on the bars in (**a**) are indicated as duplicate data points for the different treatments. Means of *RC/CSm*, *TRo/CSm*, and *DIo/CSm* are significantly different between BA and VA rates (*p* ≤ 0.05) when followed by different lowercase letters. Means of *ABC/CSm* and *ETo/CSm* are significantly different between BA and VA rates (*p* ≤ 0.05) when followed by different uppercase letters. *RC/CSm* indicates the number of active reaction centers per CS; *ABS/CSm* indicates the absorption flux per CS; *TRo/CSm* indicates the trapped energy flux per CS; *ETo/CSm* indicates the electron transport flux per CS; *DIo/CSm* indicates the non-photochemical quenching per CS.

As shown in Figure 3a, *RC/CSm, ABC/CSm*, *TRo/CSm*, and *ETo/CSm* were increased, and *DIo/CSm* was decreased by biochar and vermicompost application. BA_3_ treatment had the highest *RC/CSm*, which was significantly higher than CK by 22%, and VA_3_ and VA_5_ showed no statistically significant difference. *ABC/CSm*, *TRo/CSm*, and *ETo/CSm* of VA_3_ were significantly (*p* < 0.05) higher than other treatments (Figure 3b,c). The highest *ABC/CSm*, *TRo/CSm*, and *ETo/CSm* were observed in VA_3_ treatment, which increased by 10%, 15%, and 30%, respectively, compared with CK treatment. Meanwhile, the increase in *ABC/CSm*, *TRo/CSm*, and *ETo/CSm* in BA_3_, VA_3_, BA_5_, and VA_5_ treatments were accompanied by a decrease in *DIo/CSm*, in which VA_3_ treatment had the lowest *DIo/CSm*, with a decrease of 6% compared with CK, indicating that the application of biochar and vermicompost was effective in reducing the heat dissipation energy per cross section.

### 3.3. Yield and WUE of Tomato

Biochar and vermicompost application rates significantly (*p* < 0.05) influenced the tomatoes’ average yield and *WUE* (Table 4). The yield parameters significantly increased with the application of BA and VA rates, whereas the average single-fruit weight of the first inflorescence and second inflorescence in BA_3_, VA_3_, BA_5_, and VA_5_ was not statistically significant. In addition, the first inflorescence of each treatment was higher than that of the second inflorescence. When the *TWU* of each treatment was 1425 m^3^·hm^−2^, the WUE was linearly correlated with yield; the highest yield and *WUE* (59.0 t·hm^−2^ and 41.4 kg·m^−3^, respectively) were observed in the VA_3_ treatment, and *Y* and *WUE* under BA_3_, VA_3_, BA_5_, and VA_5_ treatments were 21%, 33%, 23%, and 25% higher, respectively, than those under CK treatment. The results indicated that the vermicompost had a better effect on increasing yield and *WUE* than biochar with the same application rates.

### 3.4. Correlation Analysis of Plant Physiological Indicators and Tomato Yield

Pearson correlation analysis results among photosynthetic parameters (*Pn*, *Ci*, *Tr*, and *Gs*), chlorophyll fluorescence traits (*Fv/Fm*, *Fv/Fo*, *ABC/CSm*, *TRo/CSm*, *ETo/CSm*, and *DIo/CSm*), and yield are displayed in Table 5. *Pn*, *Ci*, *Tr*, *Fv/Fm*, *Fv/Fo*, *ABC/CSm*, *TRo/CSm, ETo/CSm*, and yield had a strong positive correlation (*R > 0.6*), apart from *Gs*, with *ETo/CSm* and yield. These factors showed a strong negative correlation with *DIo/CSm*. In addition, photosynthetic parameters (*Pn*, *Ci*, and *Tr*) showed a strongly significant correlation with chlorophyll fluorescence traits (*Fv/Fm*, *Fv/Fo*, *ABC/CSm*, *TRo/CSm, ETo/CSm*, and *DIo/CSm*). Moreover, *Pn*, *Ci*, *Fv/Fo*, *ABC/CSm*, *TRo/CSm*, and *ETo/CSm* showed a highly significant correlation (*R > 0.8*) with yield, indicating that the increment in net photosynthesis and light energy absorbed, trapped, and transported per cross section of plant leaves could increase tomato yield.

## 4. Discussion

### 4.1. BA and VA Rates Improved Photosynthesis and Chlorophyll Fluorescence Traits

Biochar and vermicompost rates had a positive effect on photosynthetic parameters and chlorophyll fluorescence traits of treated plants. VA_3_ treatment had the highest *Pn*, *Ci*, *Fv*, *Fm*, *Fv/Fm*, *Fv/Fo*, *ABC/CSm*, *TRo/CSm*, and *ETo/CSm*, which increased by 49%, 65%, 17%, 12%, 4%, 25%, 10%, 15%, and 30%, respectively, compared with CK. Photosynthesis is the basis for crop yield and quality formation, and 95% of organic matter in crops is derived from photosynthesis [42]. Previous studies illustrated the improvement of water- and fertilizer-holding capacity of soil, enhanced photosynthesis of plant leaves, and increased *Pn*, *Tr*, and *Gs* by biochar [43,44]. Our results are supported by Cui et al. [45], who revealed that biochar significantly improved the photosynthesis of plants, and *Pn*, *Tr*, and *Gs* of 3% biochar-treated plants increased by 94%, 35%, and 35%, respectively, compared with the control. As described by Shi et al. [46], the application of vermicompost increased *Tr*, *Gs*, *Pn*, and *Ci* of tomato plant leaves by 84%, 52%, 21%, and 43%, respectively. Chlorophyll fluorescence parameters are closely related to various reaction processes in photosynthesis [47]. The energy changes in photosynthesis can be reflected by chlorophyll fluorescence-induced kinetic curves. Chlorophyll fluorescence can sensitively reflect changes in leaf photosynthesis and is a probe for studying photosynthesis [48,49,50]. Zhang et al. [51] reported that biochar reduced the shutdown of active reaction centers in alfalfa leaves, increased *Fv/Fm* of *PSII*, and enhanced photosynthesis. Fan et al. [52] showed that biochar increased *Fo* and *Fv/Fm* of plants, enhanced photosynthetic performance, and promoted plant growth and development. Yang et al. [53] found that the relative chlorophyll content of winter wheat was highly significantly and positively correlated with *RC/CSm* and *ETo/CSm*, and that *RC/CSm* and *ETo/CSm* were all related to the photosynthetic efficiency of plants, while the level of chlorophyll content reflected the strength of photosynthetic efficiency of plants and changes in fluorescence parameters [54]. Our results are also consistent with the results of Wang et al. [55], who reported that spraying exogenous phytohormones alleviated the impairment of light energy use and overall photosystem II performance in sweet potato leaves by drought stress, with good linear relationships for *Pn*, *Gs*, *Fv/Fm*, *ETo/CSm* and *ABS/CSm*. In our study, the effect of vermicompost in improving photosynthesis and chlorophyll fluorescence was more effective than that of biochar at the same application rate, as indicated by the *N* and *P* contents of the experimental vermicompost, which were 1.45 and 8.17 times higher than those of biochar (Table 1), thereby enhancing soil fertility and promoting the growth and development of tomato plants. This finding was consistent with the conclusion of previous studies, that is, vermicompost was richer in nutrients than biochar, which could remarkably enhance soil fertility [56,57].

### 4.2. Yield and WUE in Response to BA and VA Rates

The experimental results revealed significant enhancements in the yield parameters under BA- and VA-amended treatments. The application of biochar and vermicompost significantly increased the average single-fruit weight of tomatoes compared with CK (Table 4). Consequently, the total yield of each treatment was also significantly increased. Under the present experimental conditions, irrigation levels were consistent among the treatments; therefore, the BA and VA rates significantly improved *WUE*. The results are consistent with those of Blouin et al. [58], who revealed that the application of VA rates significantly increased the yield and biomass of crops. Wang et al. [59] also showed that the VA-amended treatment significantly increased tomato yield. These results were consistent with those of Zhang et al. [60] and Akhtar et al. [61], who reported that the application of biochar promoted the ability of tomato plants to absorb nutrients and increased tomato yield and *WUE*. This study also found that the application of vermicompost was more effective in improving tomato yield than biochar at the same application rate probably because the application of vermicompost under the experimental conditions increased the effectiveness of the nutrients required by the crop and promoted the photosynthesis and growth of the plant, thereby significantly increasing yield and *WUE* [62]. Our results are also supported by Ding et al. [63], who revealed that vermicompost and biochar significantly increased the yield of tomatoes every year compared with the control, and the biomass accumulation showed that vermicompost was better than biochar. Moreover, in our study, the application of 3% vermicompost (equivalent to 16,200 kg·hm^−2^) increased tomato yield by up to 32.56%, which was better than the 5% vermicompost treatment, probably because the excessive accumulation of humic acid in the high content (5%) of vermicompost inhibited plant growth and development, resulting in lower yield [64,65]. As described by Wu et al. [66], among the treatments applying different rates of vermicompost (7500, 15,000, and 22,500 kg·hm^−2^), the highest tomato yield was obtained in the 15,000 kg·hm^−2^ treatment, which was consistent with the results of our study.

## 5. Conclusions

The results showed that BA- and VA-amended treatments had a positive effect on improving photosynthetic parameters and chlorophyll fluorescence traits, particularly at the 3% application rate of vermicompost. The application of biochar and vermicompost effectively increased photosynthetic parameters (*Pn*, *Ci*, and *Tr*) as shown by studying the improvement of chlorophyll fluorescence traits (*Fv/Fm*, *Fv/Fo*, *ABC/CSm*, *TRo/CSm, ETo/CSm*, and *DIo/CSm*), producing organic substances needed by the crop, thereby increasing yield. Moreover, vermicompost with the same rate was significantly more effective in enhancing *Pn*, *Ci*, *Fv*, *Fm*, *Fv/Fm*, *Fv/Fo*, *ABC/CSm*, *TRo/CSm*, and *ETo/CSm* of plants than biochar, resulting in significantly higher yield in VA_3_ and VA_5_ than in BA_3_ and BA_5_. The *WUE* of plants under CK, BA_3_, VA_3_, BA_5_, and VA_5_ treatments increased from 31.2 to 37.8, 41.4, 38.5, and 39.2 kg·m^−3^, respectively. The results of Pearson correlation analysis revealed that biochar and vermicompost rates improved the net photosynthesis and light energy absorbed, trapped, and transported per cross section of plant leaves, which could increase tomato yield. These results indicated that vermicompost was more effective than biochar at the same rate in improving photosynthesis as shown by studying chlorophyll fluorescence and increasing tomato yield, particularly at 3% application rate.

## Figures and Tables

**Table 1:** Mean monthly climate records during the years of the experiment (2020–2021).

Season	2020						2021
**Month**	Jul	Aug	Sept	Oct	Nov	Dec	Jan
**Max. temp °C**	33.8	40.1	30.3	29.3	16.4	9.8	8.3
**Min. temp °C**	23.4	29.2	17.7	14.8	8.3	2.1	1.2
**Max. Relative humidity %**	98.6	85.0	89.5	76.0	82.3	82.6	80.2
**Min. Relative humidity %**	69.5	62.4	68.7	68.9	70.1	70.8	69.1
**Sunshine (h)**	10.2	11.4	9.1	8.3	6.5	6.1	5.8
**Solar Rad. MJ m^−2^ day^−1^**	97.8	113.7	78.2	73.8	58.7	56.0	55.4

Note: Meteorological data were monitored using HOBO mini weather stations.

**Table 2:** Specific physicochemical properties of soil, biochar, and vermicompost.

Property	Soil	Biochar	Vermicompost
***BD***	1.41 g·cm^−3^	0.42 g·cm^−3^	-
***TP***	46.3%	55.3%	-
**FC**	28.7%	-	-
***K***	101 mg·kg^−1^	58,513 mg·kg^−1^	1892 mg·kg^−1^
***N***	11.1 mg·kg^−1^	390 mg·kg^−1^	564 mg·kg^−1^
***P***	5.81 mg·kg^−1^	56.4 mg·kg^−1^	461 mg·kg^−1^
***OMC***	1.04%	41.1%	44.9%
***pH***	7.07 value	9.40 value	8.17 value

Note: Values are the average of three replicates of each property; *BD*, *TP*, FC, *K, N*, *P*, and *OMC* indicate bulk density, total porosity, field capacity, available potassium, available nitrogen, available phosphorus, and organic matter content, respectively.

**Table 3:** Effect of BA and VA rates on chlorophyll fluorescence parameters.

Fluorescence Parameters	*Fo*	*Fv*	*Fm*	*Fv/Fm*	*Fv/Fo*
**CK**	3490 ± 45 ^a^	13,253 ± 132 ^c^	16,766 ± 416 ^b^	0.791 ± 0.013 ^b^	3.80 ± 0.03 ^d^
**BA_3_**	3300 ± 86 ^bc^	15,061 ± 316 ^b^	18,390 ± 358 ^a^	0.819 ± 0.008 ^a^	4.56 ± 0.09 ^b^
**VA_3_**	3278 ± 55 ^c^	15,523 ± 203 ^a^	18,816 ± 268 ^a^	0.825 ± 0.002 ^a^	4.74 ± 0.04 ^a^
**BA_5_**	3266 ± 42 ^c^	15,095 ± 214 ^b^	18,365 ± 301 ^a^	0.822 ± 0.002 ^a^	4.62 ± 0.03 ^b^
**VA_5_**	3380 ± 63 ^b^	15,080 ± 185 ^b^	18,496 ± 240 ^a^	0.815 ± 0.001 ^a^	4.46 ± 0.03 ^c^

Note: BA for Biochar application and VA for Vermicompost application. ± indicates standard deviation. Means are not significantly different between different BA and VA rates when followed by the same lowercase letter; means are significantly different between BA and VA rates (*p ≤* 0.05) when followed by different lowercase letters. The same below.

**Table 4:** Effects of BA and VA rates on tomato yield and water use efficiency.

Treatments	Average Fruit Weight Per Fruit (g)		Yield Per Plant (g)	Yield (t·hm^−2^)	*WUE* (kg·m^−3^)
	First Inflorescence	Second Inflorescence			
**CK**	135 ± 17 ^b^	112 ± 4 ^b^	990 ± 76 ^c^	44.5 ± 3.4 ^c^	31.2 ± 2.4 ^c^
**BA_3_**	150 ± 14 ^ab^	149 ± 4 ^a^	1196 ± 68 ^b^	53.8 ± 3.1 ^b^	37.8 ± 2.1 ^b^
**VA_3_**	173 ± 12 ^a^	155 ± 18 ^a^	1312 ± 63 ^a^	59.0 ± 2.8 ^a^	41.4 ± 2.0 ^a^
**BA_5_**	157 ± 23 ^ab^	148 ± 8 ^a^	1220 ± 77 ^ab^	54.9 ± 3.5 ^ab^	38.5 ± 2.4 ^ab^
**VA_5_**	169 ± 14 ^a^	141 ± 7 ^a^	1240 ± 48 ^ab^	55.8 ± 2.2 ^ab^	39.2 ± 1.5 ^ab^

Note: BA for Biochar application and VA for Vermicompost application. ± indicates standard deviation. Means are not significantly different between different BA and VA rates when followed by the same lowercase letter; means are significantly different between BA and VA rates (*p* ≤ 0.05) when followed by different lowercase letters. The same below.

**Table 5:** Correlation analysis between plant physiological indicators and tomato yield.

Indices	*Pn*	*Ci*	*Tr*	*Gs*	*Fv/Fm*	*Fv/Fo*	*ABS/CSm*	*TRo/CSm*	*ETo/CSm*	*DIo/CSm*	*Yield*
***Pn***	1										
***Ci***	0.960	1									
***Tr***	0.918	0.936	1								
***Gs***	0.660	0.759	0.696	1							
***Fv/Fm***	0.899	0.895	0.869	0.626	1						
***Fv/Fo***	0.939	0.975	0.932	0.663	0.911	1					
***ABS/CSm***	0.950	0.947	0.903	0.635	0.829	0.949	1				
***TRo/CSm***	0.962	0.964	0.925	0.661	0.857	0.961	0.969	1			
***ETo/CSm***	0.904	0.905	0.886	0.463	0.854	0.943	0.883	0.900	1		
***DIo/CSm***	−0.775	−0.816	−0.871	−0.608	−0.795	−0.860	−0.729	−0.774	−0.859	1	
***Yield***	0.876	0.826	0.775	0.485	0.725	0.821	0.843	0.817	0.800	−0.686	1

Note: Pearson correlation coefficient ranging from 0.8 to 1.0 indicates very strong correlation, from 0.6 to 0.8 indicates strong correlation, from 0.4 to 0.6 indicates medium correlation, from 0.2 to 0.4 indicates weak correlation, and from 0.0 to 0.2 indicates very weak correlation or no correlation. “-” represents a negative correlation.

## Data Availability

Not applicable.

## References

[1] Zhou J.B., Zhai B.N., Chen Z.J., Ma A.S., Shang H.B. (2004). Spatial accumulation and potential environmental effects of soil nutrients in protected vegetable fields. J. Agric. Environ. Sci..

[2] Han P., Bayram Y., Shaltiel-Harpaz L., Sohrabi F., Saji A., Esenali U.T., Jalilov A., Ali A., Shashank P.R., Ismoilov K. (2019). Tuta absoluta continues to disperse in Asia: Damage, ongoing management and future challenges. J. Pest. Sci..

[3] Jiang L.L., Wang H.Y., Zong X.J., Wang X.F., Wu C. (2022). Effects of soil treated fungicide fluopimomide on tomato (*Solanum lycopersicum* L.) disease control and plant growth. Open Life Sci..

[4] Fan S.X., Cui J.X., Li D., Fu L.T., He X.Y., Wen J. (2021). Effects of different improvement measures on soil fertility and tomato quality in vegetable facilities. J. Agric. Eng..

[5] Zhang Y.W., Zhao P.T., Li J.M., Zhao X.G., Shang Y., Zhang Z.L., Zhao Z.P., Li L.H. (2022). Changes in photosynthetic characteristics of black wheat and their effects on yield. Chin. Agron. Bull..

[6] Fu L., Bai X.M., Yang X.H., Wu Y.X., Ai X.Z. (2013). Photosynthetic characteristics of grafted peppers and their effects on yield and quality. J. Hortic..

[7] Yang G.D., Zhou B.L., Fu Y.W., Zhang E.P., Li M. (2004). Photosynthetic characteristics, dry matter distribution and their effects on yield in different population structures of aubergine. J. Hortic..

[8] Zhu W.J., Zheng M.J., Kang Y.G. (2022). Effects of different light intensities on photosynthesis of three lianas. Chin. Agron. Bull..

[9] Zhang K., Zhang B., Wang R.Y., Wang H.L., Zhao H., Zhao F.N., Qi Y., Chen F. (2021). Effects of elevated CO_2_ concentration on photosynthesis and water physiological and ecological characteristics of spring wheat in semi-arid areas. J. Ecol. Environ..

[10] Sun M., Ma D.Y., Ji L.J., Hu H.Y., Ding X.L., Wang Z.P. (2017). Effects of different nutrient supply on photosynthesis and fruit growth and development of “Rose Fragrance” grapes. North. Hortic..

[11] Palansooriya K.N., Ok Y.S., Awad Y.M., Lee S.S., Sung J.K., Koutsospyros A., Moon D.H. (2019). Impacts of biochar application on upland agriculture: A review. J. Environ. Manag..

[12] Hang M., Liu D., Sun S.Q., Yang X.Y., Chen Q.Q., Zhang T., Lan J.Y. (2015). Effect of biochar on photosynthetic characteristics of cotton in different continuous cropping cotton field. Hubei Agric. Sci..

[13] Gui L.Q., Zhang Y.L., Wang Y.J. (2020). Research advance on effects of biochar on soil fertilityand crop’s yield and quality. Mod. Agric. Sci. Technol..

[14] Zhu Y.J. (2016). Study on the Effect of Biochar Substrate on the Growth of Tomato and Rape. Master’s Thesis.

[15] Cao X.N., Meng J., Yang T.X., Gao X., Chen W.F. (2018). Effect of biochar on cherry tomato fruit quality and yield. Jiangsu Agric. Sci..

[16] Lu J.J., Gao C.H., Li J.H., Jin D.S., Lu C.D., Dong Y.Z. (2017). Effect of straw biochar on soil nutrients and corn growth in Loess Area. Chin. Agron. Bull..

[17] Zuo Y.N., Zhang J.X., Zhao R., Dai H.Y., Zhang Z.H. (2018). Application of vermicompost improves strawberry growth and quality through increased photosynthesis rate, free radical scavenging and soil enzymatic activity. Sci. Hortic..

[18] Hosseinzadeh S.R., Amiri H., Ismaili A. (2015). Effect of vermicompost fertilizer on photosynthetic characteristics of chickpea (*Cicer arietinum* L.) under drought stress. Photosynthetica.

[19] Zhao C.R., Shan S.L., Wang Y.M., Luo K., Zhou X.F., Huang M., Zhang H.D., Fan L., Cao F.B., Chen J.N. (2017). Effects of earthworm manure application on rice growth characteristics and yield under different nitrogen levels. Chin. Rice..

[20] Zhou D.X., Shen X.Q., Zhou L.R., Cui L.J. (2012). Effect of vermicompost on agronomic characters and quality of tomato. J. Northeast Agric. Univ..

[21] Joshi R., Vig A.P. (2010). Effect of vermicompost on growth, yield and quality of tomato (*Lycopersicum esculentum* L.). Afr. J. Basic Appl. Sci..

[22] Geng D., Shan L., Li Y. (2014). Effect of soil water stress on chlorophyll fluorescence and antioxidant enzyme activity in reaumuria soongorica seedling. Chin. Bull. Bot..

[23] Mauro R.P., Agnello M., Distefano M., Sabatino L., Primo A.S., Leonardi C., Giuffrida F. (2020). Chlorophyll Fluorescence, Photosynthesis and Growth of Tomato Plants as Affected by Long-Term Oxygen Root Zone Deprivation and Grafting. Agronomy.

[24] Groher T., Schmittgen S., Fiebig A., Noga G., Hunsche M. (2018). Suitability of fluorescence indices for the estimation of fruit maturity compounds in tomato fruits. J. Sci. Food Agric..

[25] Li Z.X., Li R.J., Mu J., Yang Z.L., Sun S., Yan Y., Wang G.Y. (2020). Effects of biochar on physiological characteristics of cucumber seedlings in diethyl hexyl phthalate contaminated soil. Plant Physiol. J..

[26] Cheng H.T., Li Q.L., Liu J.K., Yan T.L., Zhang Q.Y., Wang J.C. (2017). Effects of earthworm dung soil composite matrix on the growth and chlorophyll fluorescence characteristics of Leonurus japonicus seedlings. J. Trop. Crops.

[27] Gong Z.N., Fan Y.B., Liu H., Zhao W. (2016). Chlorophyll fluorescence response characteristics of typical emergent plants under different total nitrogen gradient. Chin. Bull. Bot..

[28] Liu M.L., Liu X.Y., Pan G.X. (2017). Advance in effect of biochar on plant phenotype and gene expression. Plant Nutr. Fert. Sci..

[29] Gong Z. (2001). Chinese Soil Taxonomy.

[30] Tandon H. (1993). Methods of Analysis of Soils, Plants, Waters and Fertilizers.

[31] Sims J.R., Jackson G.D. (1971). Rapid analysis of soil nitrate with chromotropic acid 1. Soil Sci. Soc. Am. J..

[32] Henriksen A., Selmer-Olsen A. (1970). Automatic methods for determining nitrate and nitrite in water and soil extracts. Analyst.

[33] Jaiswal P. (2011). Soil, Plant and Water Analysis.

[34] Sommers D.W., Nelson L.E. (1996). Total carbon, organic carbon, and organic matter. Methods of Soil Analysis.

[35] Marx E., Hart J., Stevens R. (1999). Soil Test Interpretation Guide EC 1478 Extension & Station Communications.

[36] Li J.M., Wang P., Li J. (2010). Effects of irrigation amount on physiology, biochemistry and quality of greenhouse tomato under sub-low temperature. Agric. Eng..

[37] Farouhar G.D., Sharkey T.D. (1982). Stomatal conductance and photosynthesis. Annu. Rev. Plant Physiol..

[38] Strasser R.J., Tsimillimichael M., Qiang S., Goltsev V. (2010). Simultaneous in vivo recording of prompt and delayed fluorescence and 820-nm reflection changes during drying and after rehydration of the resurrection plant Haberlea rhodopensis. Biochim. Biophys. Acta.

[39] Yang C., Zhang Z., Gao H., Liu M., Fan X. (2014). Mechanisms by which the infection of *Sclerotinia sclerotiorum* (Lib.) de Bary affects the photosynthetic performance in tobacco leaves. BMC Plant Biol..

[40] Liu Q.Q., Ma S.B., Feng X.H., Sun Y., Yi Y.J., Liu W.X. (2016). Effects of grafting on the dynamic characteristics of rapid chlorophyll fluorescence induction of Pepper Seedlings under high and low temperature stress. Acta Hortic. Sin..

[41] Ouda S., El-Mesiry T., Gaballah M. (2007). Increasing water use efficiency for wheat grown under water stress conditions. J. Appl. Sci. Res..

[42] Zhang Y.W., Zhao X.G., Guan Z.B., Hou J.L., Wang X.F., Dong Y.H., Tian J.H., Li D.R., Wang Z.Y. (2019). Research progress on the screening of high light efficiency germplasm of crops. Chin. Agron. Bull..

[43] Liu H.M., Zhang S.Y., Guo H.G., Lin J.J., Li Z.T., Yan F.C., Zhu G.S., Xu J.Y., Zhao C.J. (2020). Effects of biochar on Millet seedling growth and photosynthetic characteristics. Agric. Res. Arid. Areas.

[44] Alfadil A.A., Xia J.H., Shaghaleh H., Hamoud Y.A., Ibrahim J.N., Hamad A.A.A., Rahim S.F., Sheteiwy M.S., Wu T.N. (2021). Wheat straw biochar application improves the morphological, physiological, and yield attributes of maize and the physicochemical properties of soil under deficit irrigation and salinity stress. J. Plant Nutr..

[45] Cui Q., Xia J.B., Liu J., Yang H., Peng L. (2020). Effects of biochar and EM bacteria on the growth and photosynthetic characteristics of Sesbania in saline alkali soil of the Yellow River Delta. J. Appl. Ecol..

[46] Shi J.W., Wang Y.D., Guan Z.H., Yao L., Lu Y.Q., Zhang Y.Q., Yang L.J. (2020). Regulation of Vermicompost on Photosynthetic Characteristics and yield of greenhouse tomato leaves. China Soil Fertil..

[47] Xia L., Zhao R., Wang Y.Q., Jin H.Y., Wu X.D., Ge J.Z., Zang F.Y., Li Z.F., Wang J.L. (2019). Effects of drought stress on photosynthesis and chlorophyll fluorescence characteristics of summer maize Effects of drought stress on photosynthesis and chlorophyll fluorescence characteristics of summer maize. J. North China Agric..

[48] Hu W.H., Yu J.Q. (2001). Effect of low temperature and low light on photosynthesis and chlorophyll fluorescence parameters of tomato leaves. J. Hortic..

[49] Luo J., Zhang M.Q. (2000). Effects of water stress on chlorophyll a fluorescence kinetics of different sugarcane varieties. J. Fujian Agric. Univ..

[50] Zhao L.Y., Deng X.P., Shan L. (2005). The osmotic stress on chlorophyll fluorescence parameters of wheat seedlings Effects of osmotic stress on chlorophyll fluorescence parameters of wheat seedlings. J. Appl. Ecol..

[51] Zhang J.H., Wu B.W.R., Wang G.L., Jia C.L., Zhang Q.P. (2018). Effect of biochar application on PS II photochemical characteristics of alfalfa leaves. Shandong Agric. Sci..

[52] Fan L.L., Muhammad w.K.T., Zhang Y., Wu X., Rong J., Zheng Y. (2021). Effects of different biochar treatments on Photosynthetic fluorescence characteristics of Fujian cypress. J. Cent. South Univ. For. Technol..

[53] Yang C., Du S.M., Zhang D.Q., Li X.D., Shi Y.H., Shao Y.H. (2021). A method for estimating the relative chlorophyll content of wheat leaves based on chlorophyll fluorescence parameters for estimating the relative chlorophyll content of wheat leaves. J. Appl. Ecol..

[54] Xu L., Gao Z.Q., An W., Li Y.L., Jiao X.F., Wang C.Y. (2016). The Photosynthetic characteristics of flag leaf under spring sowing conditions in winter wheat Changes in photosynthetic characteristics and chlorophyll fluorescence parameters of flag leaves under spring sowing conditions and their relationship with yield. J. Appl. Ecol..

[55] Wang J.Q., Li H., Liu Q., Zeng L.S. (2020). Effects of exogenous plant hormone spraying on physiological characteristics and yield of sweet potato under drought stress. J. Appl. Ecol..

[56] Wang M.Y., Jing D.W., Zhang H., Li S.P., Zheng F. (2016). Effects of earthworm manure on active organic carbon and microbial activity of cowpea soil. J. Nucl. Agric..

[57] Dong L.L., He J.Q., Lu C.Y., Shi L.L., Zhou X.W., Tao Y.Y., Wang H.H., Shen M.X. (2021). Effect of biomass carbon combined with earthworm manure on soil organic carbon and rice growth. China Soil Fertil..

[58] Blouin M., Barrere J., Meyer N., Lartigue S., Barot S., Mathieu J. (2019). Vermicompost significantly affects plant growth: A meta-analysis. Agron. Sustain. Dev..

[59] Wang Y.F., Wang C.C., Wu Z. (2020). Effects of sheep manure and earthworm manure on Agronomic Characters and quality of tomato. North. Hortic..

[60] Zhang R.H., Lan C.J., Liu W., Jin Q., Guo Y., Yu J.H., Yin L.Y., Li C.J., Huang J.Q. (2019). Effect of biochar on growth, yield and quality of open-field cherry tomato in counter season. Mol. Plant Breed..

[61] Akhtar S.S., Li G.T., Andersen M.N., Liu F.L. (2014). Biochar enhances yield and quality of tomato under reduced irrigation. Agric. Water Manag..

[62] Teng M.J., Wan B.B., Wang D.S., Jiao J.G., Liu M.Q., Chen X.Y. (2017). Effects of vermicompost application modes on growth of two tomato cultivars and soil fertility. Soil.

[63] Ding S.P., Zhang G.X., Yao Y.T., Sun Y.S., Ding F.J. (2021). Effects of combined application of earthworm manure biochar on growth and Photosynthesis of protected tomato in saline alkali land. North. Hortic..

[64] Maccarthy P., Claap C.E., Malcolm R.L. (1990). Humic Substances in Soil and Cropsciences.

[65] Brown G. (1995). How do earthworms affect microfloral and faunal community diversity?. Plant Soil.

[66] Wu J., Li J.Y., Liu N. (2018). Effects of earthworm manure organic fertilizer on tomato yield, quality and soil chemical properties. Shanghai J. Agric..

